# The enzyme profiles in the connective tissue attaching pin bones to the surrounding tissue is specific in farmed salmon (*Salmo salar*) and cod (*Gadus morhua L*.)

**DOI:** 10.1007/s10695-016-0264-9

**Published:** 2016-07-09

**Authors:** Tram T. Vuong, Sissel B. Rønning, Svein O. Kolset, Mona E. Pedersen

**Affiliations:** 1 Nofima AS, Norwegian Institute of Food, Fisheries and Aquaculture Research, Postboks 210, 1431 Ås, Norway; 2 Department of Nutrition, Institute of Basic Medical Sciences, University of Oslo, Oslo, Norway

**Keywords:** Pin bone, Connective tissue, MMPs, Serine protease, Salmon, Cod

## Abstract

*Post mortem* storage is a necessary process for removal of pin bones without destruction of fillets, thereby avoiding volume and economic loss. However, the enzymes involved in loosening pin bones during storage have not been studied to a great extent. In this study, the activities and localization of MMPs in the connective tissue (CT) of pin bones dissected from fillet of salmon and cod were investigated. Interestingly, the enzyme activity profile in these two species was different during *post mortem* storage of fish fillets. Adding MMP inhibitor (GM6001) and serine protease inhibitor (Pefabloc) revealed different effects in the two species, suggesting different regulations in salmon and cod. In situ zymography with the same inhibitors verified MMP and serine protease activity in CT close to pin bone at early *post mortem* (6 h) in salmon. However, MMP inhibition was not evident in cod in this area at that time point. Immunohistochemistry further revealed MMP9 and MMP13 were located more to the outer rim of CT, facing the pin bone and adipose tissue, while MMP7 was more randomly distributed within CT in salmon. In contrast, all these three MMPs were randomly distributed in CT in cod. In summary, our study reveals different MMP enzyme profiles in salmon and cod in the pin bone area, influenced by serine proteases, and suggests that MMPs and serine proteases must be taken in consideration when studying the conditions for early pin bone removal.

## Introduction

The consumers prefer fresh and boneless fish fillets. However, a major problem associated with pin bones is the firm attachment to the muscles which can lead to fillet destruction during removal or breakage halfway into the fillet, so that only a portion of the pin bones is removed. The fish industry needs knowledge about optimal conditions for pin bone removal to save time and costs. So far, little is known about the enzymes responsible for weakening the connective tissue (CT) surrounding the pin bones and the attachment to the surrounding tissue. There are major differences between salmon and cod in terms of bone strength and pulling force required to remove the pin bones (Akse and Tobiassen 2002; Esaiassen and Sørensen 1996; Westavik 2009). Whether this is due to a specific difference in enzymatic profiles *post mortem* in the two species is currently unknown. Degradation of the CT is enzymatic, involving numerous enzymes that can be regulated by various factors including pH, temperature and ion strength and processes that affect these factors could as such impact loosening of the pin bones (Larsen et al. 2008; Vargova et al. 2012).

Proteases are central for CT degradation and are grouped based on their catalytic residues, matrix metalloproteases (MMPs), serine proteases, cysteine proteases, threonine proteases and aspartic proteases (Cawston and Wilson 2010). MMPs are the major group of proteases important for extracellular matrix (ECM) degradation. They are classified based on their substrate specificities and include collagenases (MMPs 1, 8, 13), gelatinases (MMPs 2, 9), matrilysins (MMPs 7, 11, 26) and stromelysins (MMPs 3, 10) (see (Pedersen et al. 2015) for review of MMPs in fish). The MMPs are normally secreted as zymogens, which are subsequently processed by proteolytic enzymes to generate the active forms (Okumura et al. 1997; Woessner 1991). Under normal physiological conditions, the proteolytic activity of the MMPs is controlled at any of the following three known stages: transcription, activation of the zymogens and inhibition of the active forms by various tissue inhibitors of MMPs (TIMPs) (Verma and Hansch 2007). Extracellular proteases influence and activate each other in a complex network, and often one protease pathway is combined with another (He et al. 1989; Shamamian et al. 2001; Zhu et al. 2001).

In this study, we compared extracellular enzymes present in the attachment area of pin bones in salmon and cod during the *post mortem* period. The aim was to investigate the specific distribution of MMP activities in this specific area. Samples were harvested at different time points *post mortem*, and enzyme expression and activities around pin bones area were investigated by immunohistochemistry and in situ zymography. Our results reveal new and important distribution patterns of MMPs in salmon and cod and also differences between the two species during *post mortem* storage.

## Materials and methods

### Fish samples

Tissues were obtained from salmon (*Salmo salar*) and cod (*Gadus morhua L*.). Fillets harvested immediately after slaughter were stored on ice for 0 min, 6, 12, 24 h or 5 days. For total MMP activity assay, pin bones with surrounding CT (including some residues of surrounding adipose/muscle tissue) were dissected, snap frozen in liquid nitrogen and stored at −80 °C until further analysis. For microscopy studies, pieces including pin bone area of approximately 15 × 10 × 10 mm were cut from anterior positions in the fish fillets and fixed in zinc-buffered fixative (36.7 mM ZnCl_2_, 27.3 mM ZnAc_2_ × 2H_2_O, 0.63 mM CaAc_2_ in 0.1 M Tris, pH 7.4) for 36–38 h. Thereafter, the samples were decalcified with EDTA (14 %, pH 7.1 at RT) for 10 days, before dehydration and paraffin embedding.

### Protein extraction from pin bone tissue

The frozen pin bone tissue from salmon or cod were homogenized in 2-ml tubes with 2.8-mm ceramic beads (Precellys) and 20 µl of lysis buffer (0.25 % Triton X-100 in 10 mM CaCl_2_ and 100 mM HEPES, pH 7.0) per mg tissue using Precellys 24 tissue homogenizer at 5.000 rpm for 4 × 30 s. The tubes were then incubated on ice for 1 h before being centrifuged at 13.000 rpm for 15 min at 4 °C. Protein concentration in the supernatants was determined with BCA protein assays (Thermo Fisher Scientific) before storage at −80 °C until further analysis.

### MMP activity assay

Total MMP activity was measured using SensoLyte 520 generic MMP assay kit fluorometric (AnaSpec). In brief, equal amount (3 µg) of pin bone tissue extracts was added to a 96-well plate and diluted with assay buffer to 50 µl/well. For inhibition assay, 0.5 mM GM6001 (Calbiochem) or 8 mM Pefabloc (Roche) was added to the samples. A blank control of only assay buffer was also included. A total of 50 µl/well of the generic MMP substrate solution was added to the sample and control wells, and the reagents were mixed by gentle shaking the plate for 30 s. The plate was incubated at RT for 1 h in darkness before the fluorescence intensity was measured at Ex/Em = 490/520 nm with a fluorometric microplate reader. The MMP activity was expressed as relative fluorescence unit (RFU) by subtracting the background reading from the sample readings.

### In situ zymography

The method was performed as described in Hadler-Olsen et al. (2010). In brief, 5-µm-thick sections from ZBF-fixed and paraffin-embedded pin bone tissue (6 h) were heated at 58 °C overnight, deparaffinized in xylene and rehydrated in graded alcohol baths. Two hundred milliliters substrate of dye-quenched (DQ) gelatin (Invitrogen), DQ-collagen or DQ-casein (Life Technologies) diluted 1:50 in reaction buffer (50 mM Tris–HCl, 150 mM NaCl, 5 mM CaCl_2_, 0.2 mM sodium azide, and pH 7.6) was added to the tissue sections and incubated in dark humidity chamber at 37 °C for 2 h. To evaluate the contribution of proteases, sections were pre-incubated with 0.5 mM GM6001 or 8 mM Pefabloc for 1 h at 37 °C. Sections were then rinsed 2 × 5 min in PBS baths, dipped in Milli-Q water and air-dried for few minutes. The sections were mounted using SlowFade Gold antifade reagent with DAPI (Invitrogen) and examined with a confocal microscope Olympus FluoView FV1000 (Olympus).

### Immunohistochemistry

Sections were deparaffinized and rehydrated before permeabilizing with 0.5 % Triton-X100 in PBS for 15 min. Non-specific antibody binding was blocked by incubating the sections with 5 % BSA for 1 h. The sections were incubated with mouse anti-MMP7 (Santa Cruz), rabbit anti-MMP9 (Novus Biological) or rabbit anti-MMP13 (Abcam) antibodies (all diluted 1:50) for 2 h, before washing with PBS and subsequently incubation with Alexa 546-conjugated secondary antibodies (Invitrogen, 1:600) for 45 min. Sections were washed in PBS, mounted with SlowFade Gold antifade reagent with DAPI and examined with a confocal microscope Olympus FluoView FV1000. The images were processed using Adobe Photoshop CS4 (Adobe Systems Inc.), brightness and contrast, if used were adjusted manually across the entire image.

## Results and discussion

**Fig. 1:**
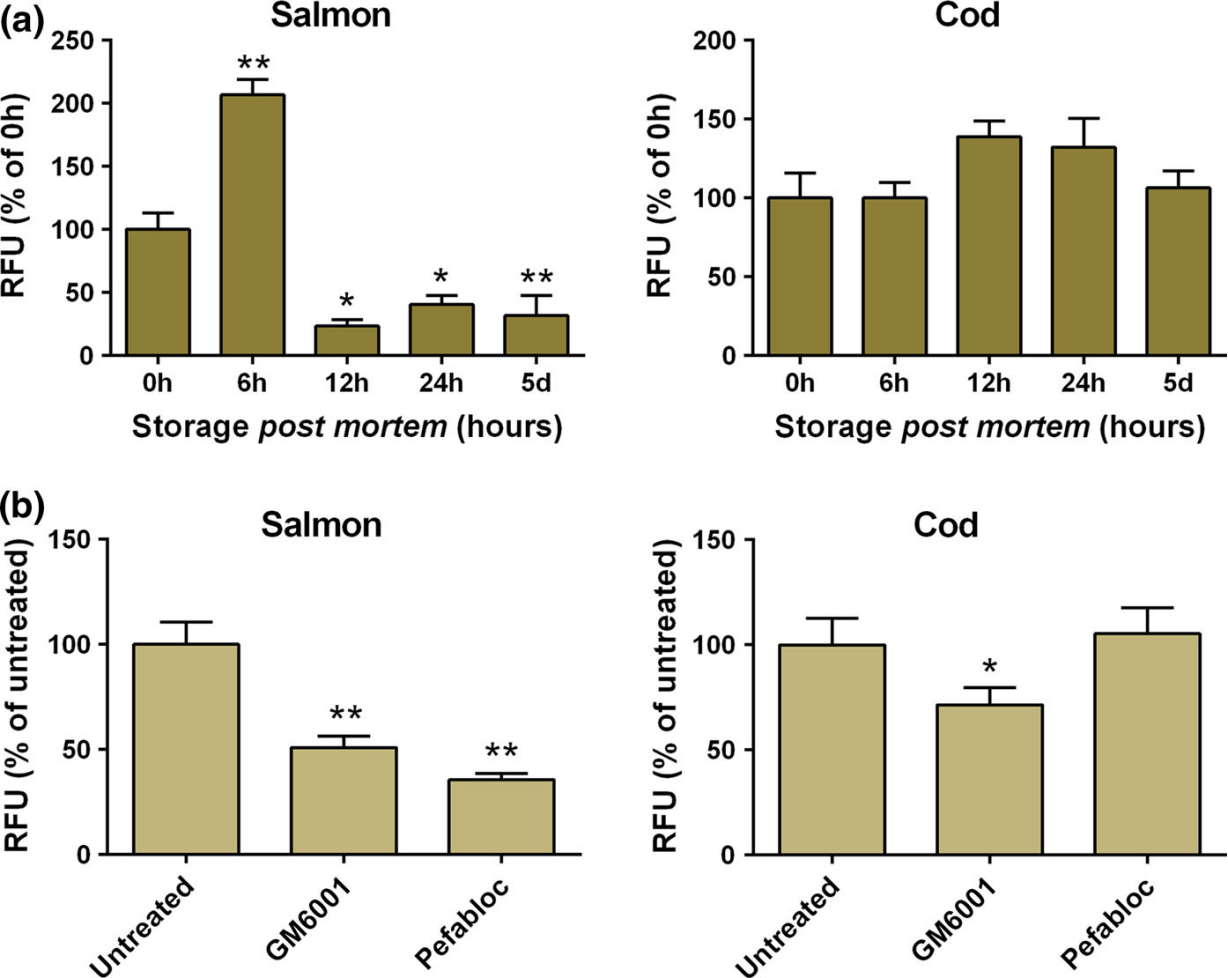
Total MMP activity in pin bone area of salmon and cod determined using generic MMP activity assay. **a** MMP activity during *post mortem* storage on ice (*n* = 10). **b** MMP activity measured in presence of the universal MMP inhibitor GM6001 and serine protease inhibitor Pefabloc (*n* = 4). The results are expressed as mean ± SEM. *p ≤ 0.05 and **p ≤ 0.01 indicate statistically significant difference between the 0 h and the storage times *post mortem*, or between untreated and inhibitor treatments, as evaluated by the Student’s unpaired *t* test

Our results show that the total MMP activity in salmon increased significantly short time after slaughter (6 h), followed by a dramatic reduction already after 12 h storage (Fig. 1a, left panel). This is in contrast to the MMP enzyme profile in cod, where the activities of MMPs sustained during storage, with just a minor increase at 12 and 24 h (Fig. 1a, right panel). To characterize the regulation of MMP activity in the two species, we added an MMP inhibitor (GM6001) and a serine protease inhibitor (Pefabloc) to the samples collected at 6 h before and we measured the MMP activity (Fig. 1b). Interestingly, our experiments demonstrate that the MMP activity was regulated differently in salmon and cod. Our results show that adding GM6001 inhibited total MMP activity in both salmon and cod. However, when adding Pefabloc, the total MMP activity was inhibited in salmon, while no effect on activity was observed in cod. This suggests that serine proteases are important for MMP activation in salmon, but not to the same degree in cod. Our unpublished proteomic data analysis (Rønning et al. 2016 under revision by Fish Physiology and Biochemistry) also shows a different protein composition and expression in salmon and cod. In addition, there are changes in a relatively large number of proteins during storage, indicating that the connective tissue surrounding pin bone is subject to multiple post mortem changes. Differences in expression of enzymes and regulatory proteins could possibly result in different regulations and kinetics in the two species.

**Fig. 2:**
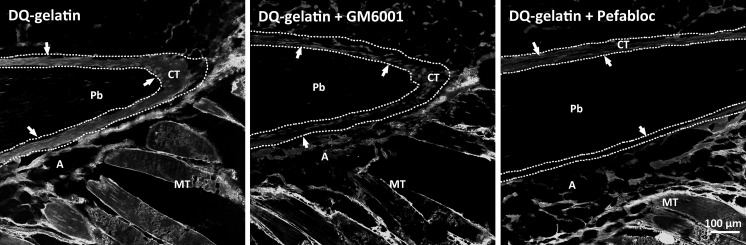
In situ zymography of salmon pin bone tissue section with DQ-gelatin in presence or absence of the universal MMP inhibitor GM6001 and the serine protease Pefabloc. *Arrows point* out the CT surrounding the pin bone. *Pb* pin bone; *CT* connective tissue; *A* adipose tissue; *MT* muscle tissue. *Scale bars* as indicated

In situ zymography visualizes the precise localization of the enzyme activities in the tissue. Our experiment with MMP substrate DQ-gelatin demonstrated the presence of MMP activity in the CT surrounding pin bones and in the surrounding muscle and adipose tissue of salmon (Fig. 2, left panel). Using GM6001 and Pefabloc, the gelatinolytic activity in the CT was inhibited (Fig. 2, middle and right panel). Using different substrates, (gelatin, collagen and casein) we demonstrated MMP activity and serine protease activity in the CT close to the pin bones, summarized in Table 1. Inhibition of the enzyme activity in the CT close to pin bone was less visible in cod (Table 1), most likely reflecting less MMP activity present at the time point studied (6 h). Interestingly, although the enzyme activity was clearly inhibited in the CT, the activity in the surrounding tissue was not depressed by MMP or serine protease inhibitors, revealing a different enzyme profile in the CT close to pin bone compared to the CT in surrounding skeletal muscle and adipose tissue. MMPs exhibit a broad range of substrate specificities, including ECM proteins as well as non-ECM proteins. Collagen and gelatin are preferred substrates for the collagenase family and gelatinase family, respectively, although they can also be cleaved by other MMPs (Nagase 2001). Casein is a less common and preferred substrate for MMPs, but are frequently used in zymography for determining activity of MMP1 and MMP7 (Hu and Beeton 2010; Snoek-van Beurden and Von den Hoff 2005; Zeng et al. 2002). Casein is also a substrate for serine proteases. Under normal physiological processes, MMPs must be expressed to the exact extra- or peri-cellular location, at the right time and in the right amount. Also, they must be activated or inhibited appropriately. Most MMPs are synthesized and secreted as inactive proenzymes, and plasmin has been described as a key activator of several MMPs (He et al. 1989; Murphy et al. 1999). However, many other serine proteases have also been shown to directly activate MMPs in vitro or in vivo (Duncan et al. 1998; Fang et al. 1997; Gruber et al. 1989; Okada et al. 1987; Shamamian et al. 2001). In all cases, the activation requires the disruption of the Cys-Zn^2+^ interaction in their active center, and the removal of the propeptide that often proceeds in a stepwise manner involving the actions both from serine proteases and from activated MMPs (Gruber et al. 1989; Shamamian et al. 2001; Zhu et al. 2001).

**Table 1:** Inhibition of GM6001 and Pefabloc on MMP activity in the pin bone connective tissue in salmon and cod with different DQ-substrates

	Salmon		Cod	
	GM6001	Pefabloc	GM6001	Pefabloc
DQ-Gelatin	++	++	−	−
DQ-Collagen	+	++	−	−
DQ-Casein	+	++	−	+

The inhibitions by GM6001 and Pefabloc were scored by visual judgment of fluorescence intensity in the CT of salmon and cod

‘+’ indicates inhibition, ‘−’ indicates no inhibition observed

**Fig. 3:**
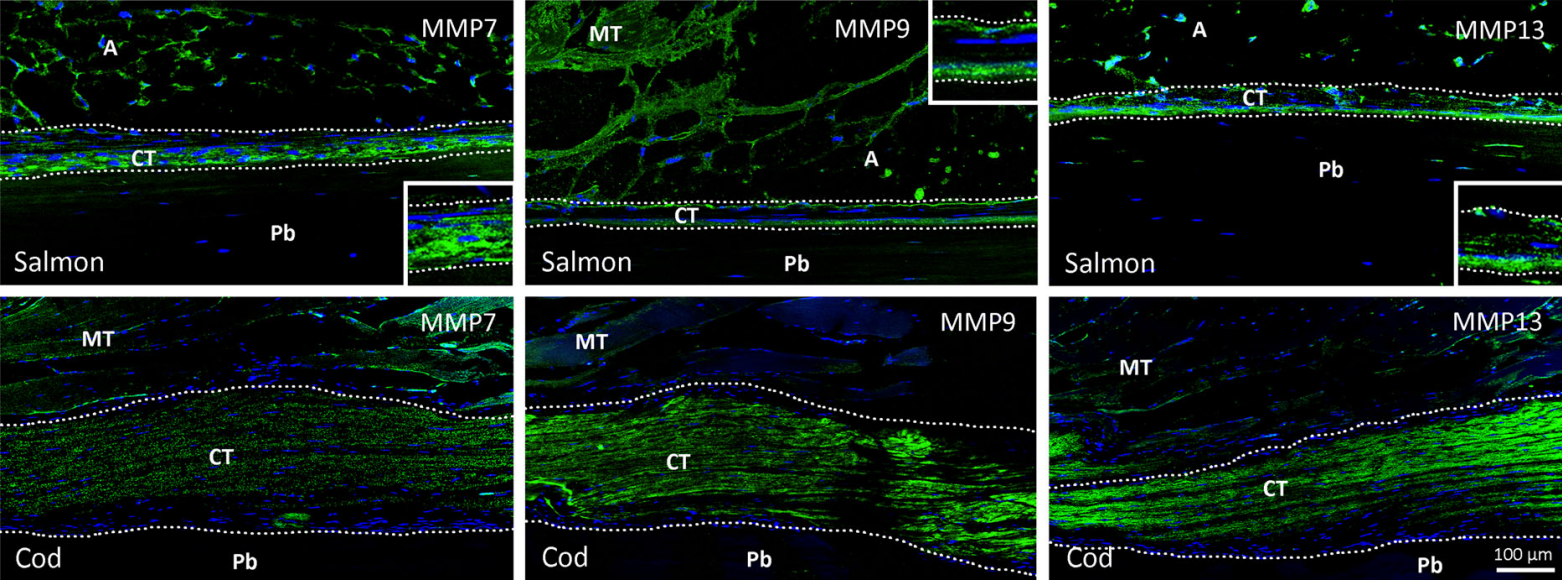
Immunofluorescence staining of pin bone tissue sections from salmon and cod with antibodies against MMP7, MMP9 and MMP13. *Insets* show magnifications of CT. *Pb* pin bone; *CT* connective tissue; *A* adipose tissue; *MT* muscle tissue. *Scale bar* as indicated

Using immunofluorescence, we demonstrate the presence of MMP7, MMP9 and MMP13 in the CT close to the pin bones (Fig. 3). In salmon, MMP9 and MMP13 were located to the outer rim of the CT, facing the pin bone and adipose tissue, while MMP7 was distributed more randomly. This is in contrast to the location in cod, where the MMPs were randomly distributed in the CT surrounding pin bones. Experiments in our laboratory have shown that the degradation of the CT in salmon and cod is different during *post mortem* storage (unpublished data). In salmon, the loosening occurred at the interface of pin bone and CT during degradation. This is in contrast to a more even degradation within the CT of cod. The different MMP distribution patterns in salmon and cod, especially MMP9 and MMP13, should be of interest for further studies. Proteolysis often occurred in the immediate vicinity of the cell in peri-cellular pockets close to the cell membrane where MMPs can be secreted to specific areas at the cell surface. Such localization mechanisms could possibly allow a high degree of control and can enhance MMP activity, prevent access of MMP inhibitors, concentrate MMPs to their precise target substrates and limit the extent of proteolysis to a defined region (Zucker et al. 2003).

There are several MMPs identified in fish, but the precise functions of these are not well characterized yet (see (Pedersen et al. 2015) for review). MMP9 and MMP13 have been detected at mRNA levels in salmon bone tissue (Ytteborg et al. 2010). In bone, the removal of the outer osteoid layer by MMPs precedes the attachment of the osteoclast and the subsequent breakdown of the ECM by cysteine proteinases (Everts et al. 1992). MMPs have been proposed to participate in degradation of fish muscle during storage (Kubota et al. 2001, 2003; Lodemel et al. 2004; Wu et al. 2008), and both MMP2 and MMP9 have been demonstrated in muscle fillet of Atlantic cod, spotted wolfish and Atlantic salmon (Lødemel and Olsen 2003). In common carp, MMP2 plays a critical role in muscle softening by degradation of type I and V collagens (Xu et al. 2015). By immunohistochemistry, we could observe expression of MMP2 and MMP9 in connective tissue of skeletal muscle in our salmon and cod samples (data not shown). However, only MMP9 was detectable in the pin bone tissue (Fig. 3), suggesting that MMP2 is not a major contributor in softening of connective tissue surrounding pin bones. Enzyme analysis of firm and soft fillets from Atlantic salmon revealed more active MMPs in the softer muscles (Martinez et al. 2011). In addition, the presence of serine protease activity in skeletal muscle of red sea bream and hake has been demonstrated and suggested to be involved in texture tenderization of the fish muscle (Martone et al. 1991; Wu et al. 2010).

## Conclusion

In this study, we have compared extracellular enzymes present in the attachment area of pin bones in salmon and cod during the *post mortem* periods. Our results demonstrate that salmon and cod have a different enzyme profile, with a different distribution of MMPs in the CT. Further, we show that there is a complex network of MMPs and serine proteases influencing each other, making both MMPS and serine proteases interesting targets for further studies optimizing the early pin bone removal process in salmon industry.
